# Maximal Intraoperative 5-Aminolevulinic Acid Fluorescence During Surgical Resection of Multiple Lesions in Multifocal Glioblastoma to Achieve Greater Extent of Resection: A Case Report

**DOI:** 10.7759/cureus.79083

**Published:** 2025-02-16

**Authors:** Javier A Jacobo, Jorge Aristizabal, Andres F Cardona-Zorrilla

**Affiliations:** 1 Neuro-Oncology, Fundacion Centro de Tratamiento e Investigación sobre Cáncer (CTIC) Luis Carlos Sarmiento Angulo, Bogota, COL; 2 Neurosurgery, Fundación Cardioinfantil – laCardio, Bogota, COL

**Keywords:** 5-ala, fluorescence, glioblastoma, multifocal, surgery

## Abstract

Multifocal glioblastoma represents a therapeutic challenge with conflicting evidence on treatment, though it appears that the extent of resection also plays an important role in this disease. The use of 5-aminolevulinic acid has been shown to improve the extent of resection in high-grade gliomas; however, there is little information about its use in multifocal glioblastoma. We present the case of a 79-year-old patient with distant glial lesions who underwent surgery for the resection of a right temporal and a right occipital lesion at the same surgical time. Intraoperative findings and postsurgical events are described in this report. This case shows that resection of multiple lesions in a patient with multifocal glioblastoma using intraoperative 5-aminolevulinic acid fluorescence is feasible and safe.

## Introduction

Diffuse gliomas are the most frequent among primary intracerebral tumors in adults. These are malignant lesions derived from glial cells in the brain and usually have dismal prognoses despite treatment with surgery, chemotherapy, and radiotherapy [1]. Surgical resection is accepted as the initial management for newly diagnosed gliomas, and extensive resection of the contrast-enhanced region in high-grade gliomas has a positive impact on the prognosis of these patients [1]. Several studies have shown a positive relation between a greater extent of resection (EOR) and a more favorable survival [2,3]. The EOR is the relation of residual tumor after surgery compared to the preoperative image, and a gross total resection (GTR) implies no residual tumor after surgery. 

Multifocal glioblastoma (mGB) represent a therapeutic challenge with conflicting evidence on treatment. Classically mGB has shown a worse prognosis when compared with unifocal lesions, and surgical approaches to multiple lesions in the same surgical timing were deemed as a great risk with minimal benefits. However, a recently published cohort by Trip et al. supports the idea that a greater EOR also plays an important role in improving survival in this disease [4]. 

In glioma surgery, several intraoperative aids have been studied to help the surgeon achieve greater EOR in high-grade gliomas (HGG). Among these, the use of intraoperative fluorescence with 5-aminolevulinic acid (5-ALA) has been shown to improve the EOR in several studies [5]. It should be noted that 5-ALA is a metabolic precursor for heme biosynthesis. Tumor cells have alterations in this metabolic chain and 5-ALA is converted to protoporphyrin IX, which accumulates in the mitochondria and can have a fluorescent effect after stimulation with light between 405 and 633 nm, and intraoperatively under the microscope, tumor tissue is visualized as a bright pink mass. Few studies are found on the use of 5-ALA in mGB, and this is a current void in the literature. For instance, in the recently published RESECT trial that included 171 patients, none of the patients harbored multifocal lesions [5]. We report the case of a patient with mGB who was taken into surgery for the resection of two lesions and document the intraoperative results.

## Case presentation

Clinical presentation

**Figure 1:**
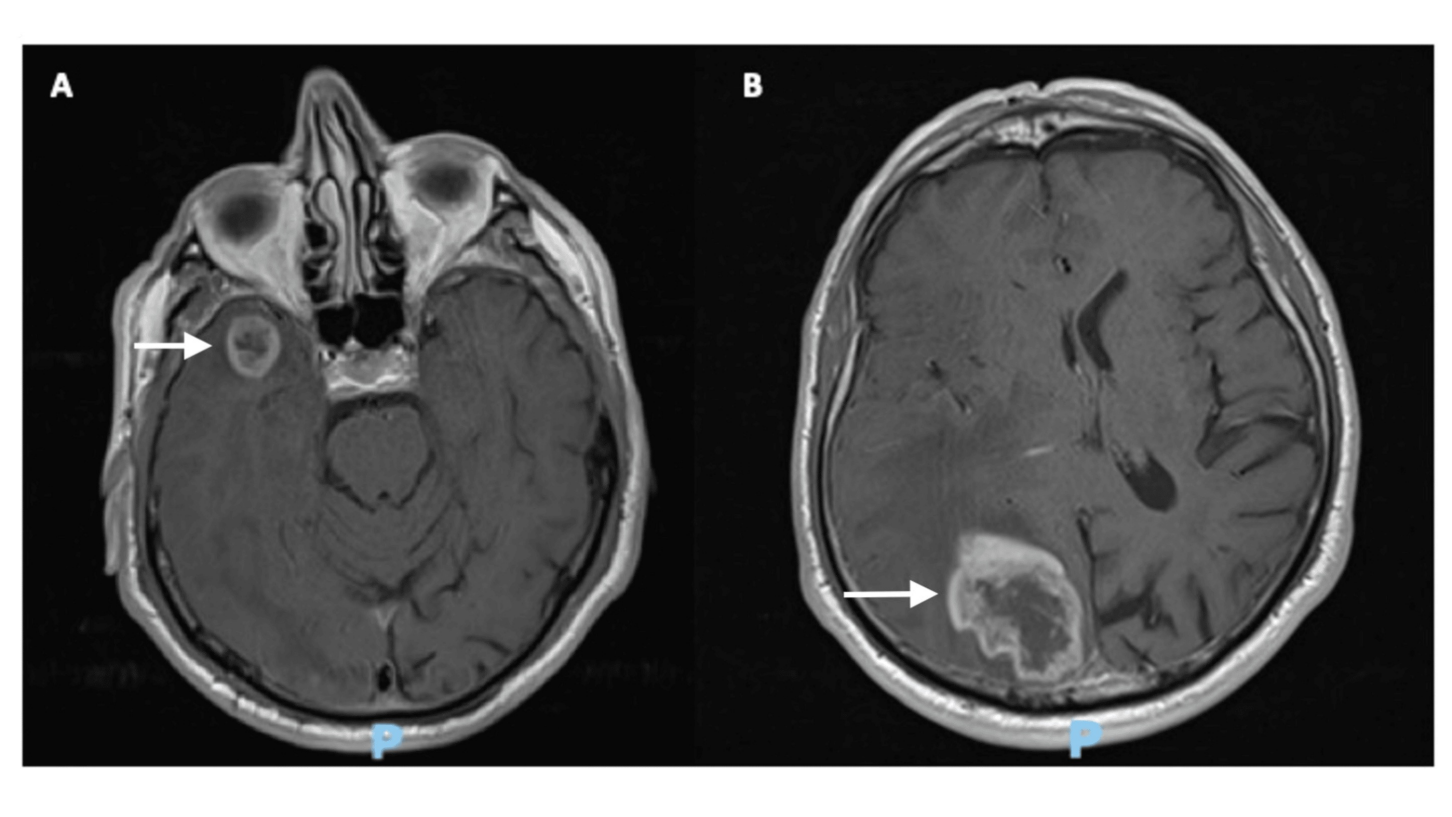
Preoperative gadolinium-contrast-enhanced T1-weighted MRI showing an enhancing lesion at the right temporal pole and the right occipital lobe (white arrows)

A 79-year-old independent male patient presented with left-sided weakness and recurrent minor head trauma due to unawareness of his left visual field. Neurological examination revealed a 4/5 left side hemiparesis and a bilateral defect on the left visual field. No sensory deficit or cognitive impairments were documented. Magnetic resonance imaging (MRI) was performed that revealed two distant lesions: the first was an intracerebral lesion approximately 3.5 cc in volume with a heterogenous signal that showed irregular contrast enhancement after gadolinium injection at the right temporal pole, and was associated with vasogenic edema. The second was an intracerebral lesion with a heterogenous signal that showed strong, irregular contrast enhancement after gadolinium injection in the right occipital lobe with a maximal diameter of 4.6 cm, showing extensive vasogenic edema that collapsed the ipsilateral ventricular system (Figure 1).

After discussion in the Neuro-Oncology Tumor Board, it was decided that the patient could benefit from surgical resection of the two lesions under the presumption that we were dealing with an mGB. As intraoperative aids, ultrasound and 5-ALA fluorescence were chosen to help achieve a greater EOR.

Surgical protocol

Assessment of renal and hepatic function was made as well as all presurgical labs. The day before surgery, corticosteroids were suspended, and antiemetic drugs were administered intravenously until the time of surgery. On the day of the surgery, 5-ALA was administered orally at a dose of 20 mg/Kg, two hours prior to anesthesia induction according to the pharmacological recommendations. It has been described that the fluorescence peak is reached at four to eight hours after administration, so we chose to administer the medication closest to the time of anesthesia induction so that we could have a fluorescence effect at the first and second craniotomies. Physical barriers were put in place to protect the patient from direct sun or fluorescent light on his way to the operating room. Anesthesia induction was carried out in standard protocol under dim lights and then the patient was draped so that the surgery could take place under normal lighting.

Intraoperative findings

**Figure 2:**
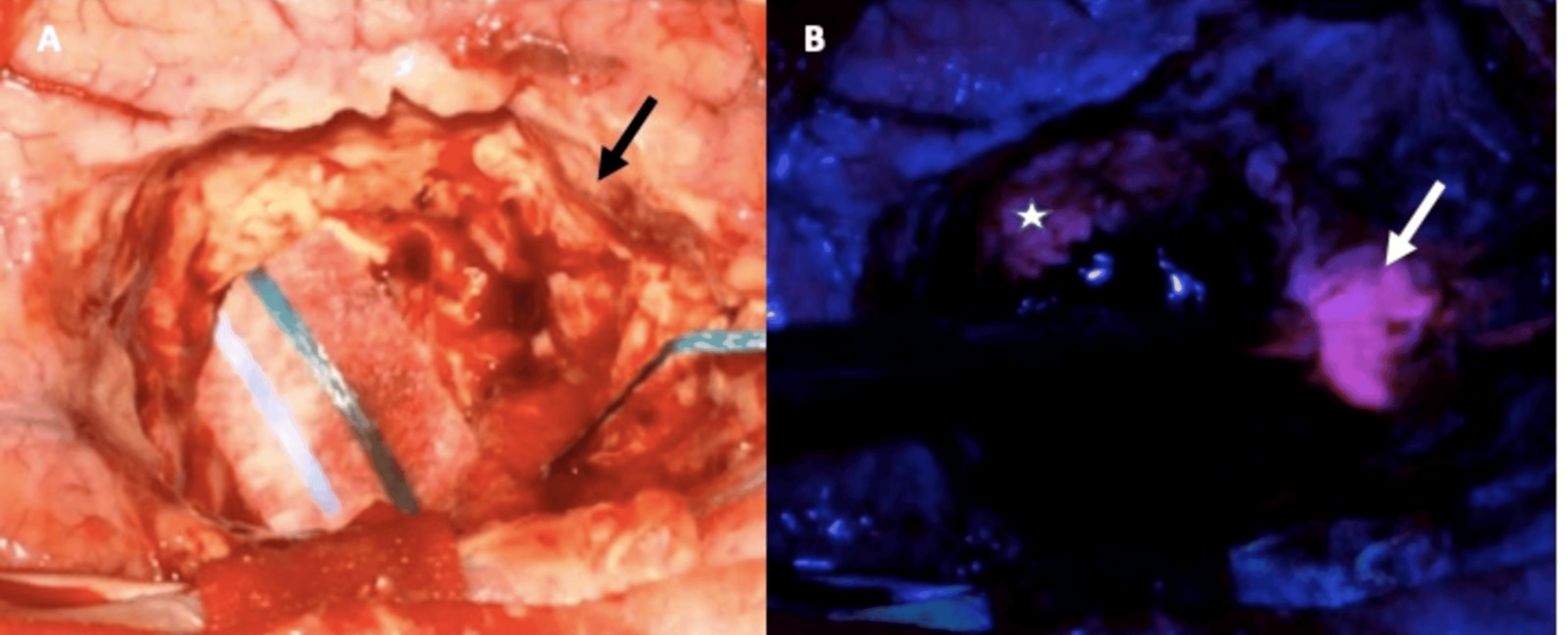
Intraoperative findings for the occipital lesion (A) Surgical cavity under white light shows normal glial tissue at the borders (black arrow). (B) Under the blue light filter, a residue is found with strong fluorescence (white arrow), and a less strong fluorescence is shown at the limits of the surgical cavity indicating infiltrative tumor tissue (white star). The less intense fluorescence is related to tumor infiltration beyond the limits of the contrast enhancement in the MRI and helps achieve supratotal resection in some described cases [5].

Occipital craniotomy was carried out at the beginning of the surgery. Exposure of the tumor was made close to four hours after 5-ALA administration. The tumor exhibited strong pink fluorescence (Figure 2) which served as an aid to achieve GTR. After occipital wound closure, the right temporal region was exposed.

**Figure 3:**
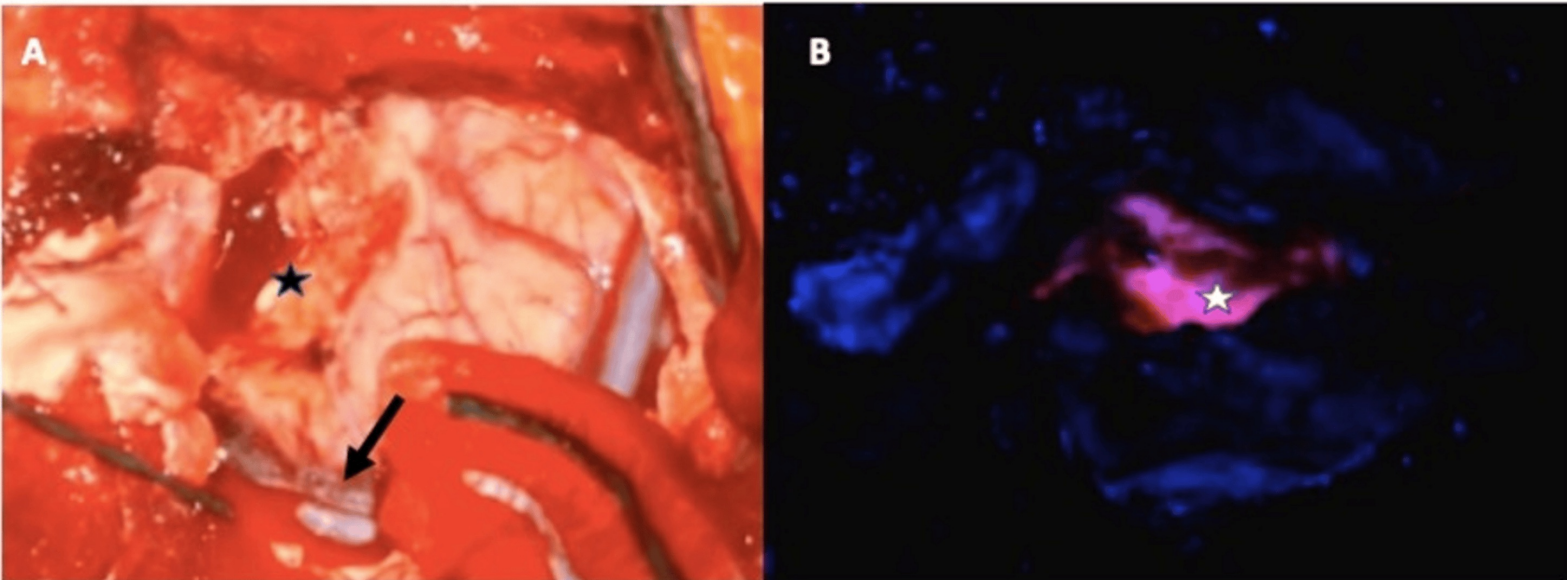
Intraoperative view of the temporal lesion (A) A view of the temporal lobe under white light. The black arrow points at the superficial Sylvian vein. The black star is located at the initial corticotomy near the temporal pole. (B) Surgical cavity under blue light. The white star is located at the surgical cavity showing a strong fluorescence by the high-grade tumor.

A right temporal craniotomy was performed, and the temporal pole lesion was exposed at the six-hour mark after 5-ALA administration. The temporal lesion also showed a strong pinkish fluorescence and resection of the lesion was carried out until this fluorescence was no longer visible (Figure 3). Closure of the temporal wound was carried out under normal circumstances.

Postoperative period

**Figure 4:**
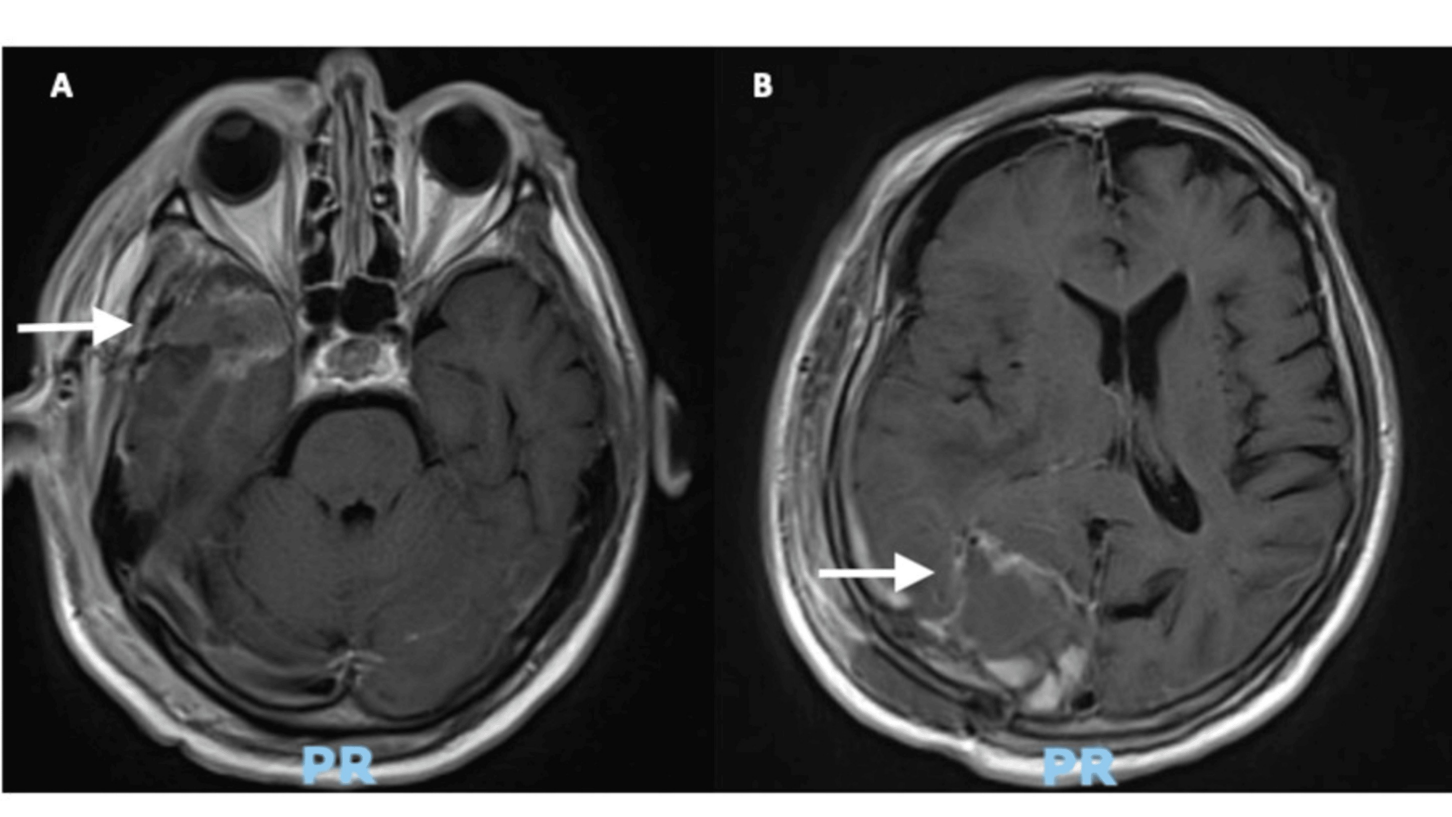
Postoperative MRI showing complete resection of the temporal (A) and occipital (B) lesions.

The patient recovered from surgery with no additional neurological deficits or complications and was discharged after three days. Postoperative MRI at 24 hours after surgery showed no visible residual tumor in either lesion (Figure 4). The pathology results for both lesions informed the presence of an IDH1 wild-type glioblastoma.

## Discussion

The role of surgery in the management of mGB is controversial [4,6]. The overall survival of patients harboring mGB is generally short, with estimates being made at around three to nine months after initial diagnosis [6-8]. Although the evidence suggests that achieving a GTR could affect the prognosis in a positive way, with a median survival of 13 months in cases of mGB after GTR [6]. The benefits of surgical resection in mGB could be extended to better tolerate adjuvant treatment as well because of the reduction of the tumor load and mass effect [9]. Our patient went on to receive adjuvant treatment with hypofractionated radiotherapy with adequate tolerance.

Given that a greater EOR influences the prognosis of patients with HGG and mGB, it is important to take steps to achieve this goal. Intraoperative fluorescence with 5-ALA has been shown to improve EOR in patients with HGG [5,10]; however, there is little information regarding the use of this method of fluorescence in multiple lesions. In a recent case series by Strickland et al., the authors described 30 patients treated using angled endoscopic blue light visualization achieving a mean EOR of 90.7% [11]. Among the treated patients, one patient had a multifocal HGG; however, in this case, the authors only performed a biopsy. We have not been able to find documentation of simultaneous resection of multiple lesions in HGG using 5-ALA. In the presented case, we were able to achieve a GTR in both lesions. This, however, should be considered indicative of a trend that can be observed in every case of mGB that is operated using 5-ALA fluorescence as an intraoperative aid. 

The use of 5-ALA in the surgical resection of intracranial tumors has some technical nuances that must be considered in order to fully take advantage of its fluorescence properties. For instance, the time duration from the administration of the molecule and the time of tumor resection must be calculated so that the maximum grade of fluorescence of 5-ALA is manifested during tumor resection, at about six to eight hours after administration [5]. This timing window is of great importance especially when attempting surgery for multiple lesions, since false negative fluorescence is usually the result of technical issues, and performing surgery after this time period could result in the absence of fluorescence even in the presence of a high-grade lesion [12]. Having this time window in mind, we administered the 5-ALA closer to the time of surgery so that we could perform the two sequential craniotomies and still have sufficient fluorescence to perform the tumor resection and showed that it was possible to achieve strong fluorescence in both tumors. GTR of both lesions was confirmed in the postoperative MRI, but more importantly, the patient had no additional deficits after surgery.

When performing surgery in patients with mGB, the postoperative functional status must be considered, since it is one of the most important prognostic factors for these patients [13]. Studies have shown that resection of multiple lesions in patients with mGB does not translate into worse neurological status and should not be a factor in deciding whether or not to operate in these patients [9]. In addition to the multicentric nature of the patient’s tumor, the patient’s age needs to be discussed, since doing extensive surgery on a patient over 70 years of age could be presented as controversial. However, the available data suggests that patients over 65 years of age with excellent functional status should be considered for standard treatment consisting of surgery and radiotherapy with adjuvant temozolomide [14]. It needs to be stated that aggressive brain surgery in elderly patients carries additional risks when compared to younger patients; however, all cases should be assessed individually, since variables like obesity and the frailty index are independent factors for worse prognosis in elderly patients [15]. In the case we present, this 79-year-old patient had no medical comorbidities, and his functional status was optimal, which is why we decided on an aggressive treatment option. 

## Conclusions

Resection of multiple lesions in a patient with mGB using intraoperative 5-ALA fluorescence is feasible and safe. Following recommendations, it is possible to achieve maximal fluorescence in multiple distant lesions with a single administration close to the timing of surgery. We hope that the results shown in this report encourage the implementation of this protocol in a larger cohort so that these conclusions can be further validated. It is important to highlight that this is a single case and these results should be interpreted with care and each case should be discussed individually. 
